# Supplementary material for: Thymosin Beta 15 Alters the Spatial Development of Thymic Epithelial Cells
Source: Cells. 2022 Nov 19;11(22):3679. doi: 10.3390/cells11223679 (PMC9688846; doi:10.3390/cells11223679)
Supplement: Supplementary file 1 [file cells-11-03679-s001.zip › cells-2008225-supplementary.pdf]

cells

1. Supplementary Material

1.1. Supplementary Figure Legends

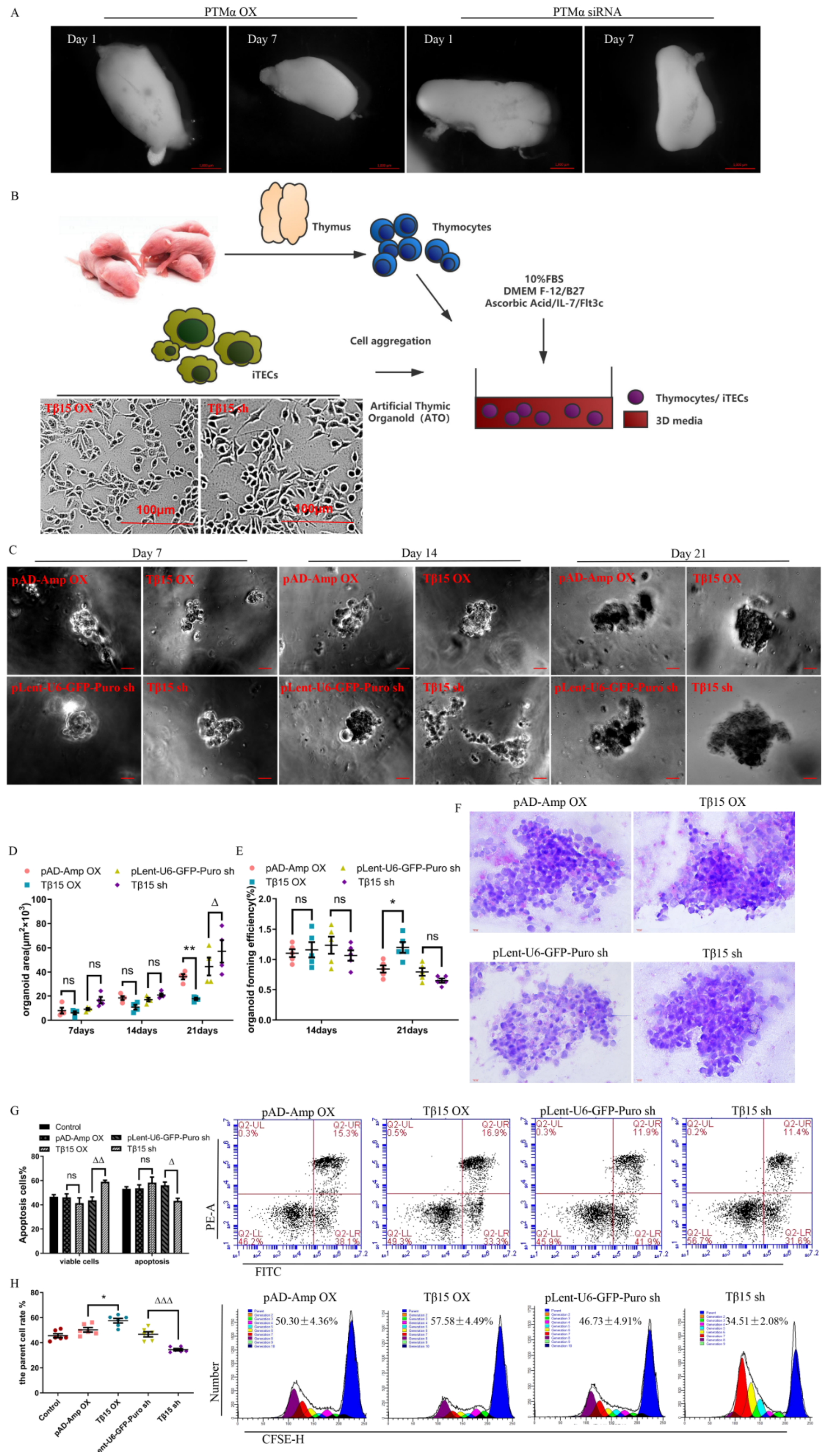

Supplementary Figure S1. Tβ15 inhibites iTECs-mediated proliferation of thymocytes in early stage in 3D organoids cultures. (**A**) Representative images of the thymus tissues transduced with PTM*α*

siRNA or PTMα OX for 7 days. Scale bar = 1000 µM. (**B**) Schematic of 3D organoid co-culture methods. Tβ15-transduced iTECs are placed in co-culture with isolated murine thymocytes in hyaluronic acid hydrogel. Representative images of the different stromal cells were shown in the lower panel. Scale bar = 100 µM. (**C**) Representative images of organoids formed from 3D co-culture of thymocytes with iTECs after 7, 14, 21 days. Scale bar = 20 µM. (**D**) Organoid size measured on pictures from the indicated co-cultures for 7, 14 and 21 days. Each symbol represents thymic organoid volume per group (n=4). (E) Quantification of number of thymic organoids formed in co-culture after 14, 21 days. Data represent the organoid forming efficiency (%). (**F**) Representative images of thymic organoids stained with H&E. Scale bar = 10 µM. (**G**) Flow cytometry using Annexin V-FITC staining shows that stable knockdown of Tβ15 increases iTECs-mediated apoptosis of thymocytes. Data represent the percentages of alive and apoptotic cells. (**H**) Thymocytes labeled with CFSE are analyzed for proliferation after Tβ15 treatment. Data represent the parent cell rate (%). All of the data are from at least 3 independent experiments and error bars represent SEM, A one-way ordinary ANOVA test was used in H; two-way ANOVA test was used in D, E and G. * $p < 0.05$, ** $p < 0.01$,*** $p < 0.001$ compared to pAD-Amp OX group. $^{\#} p < 0.05$, $^{\#\#} p < 0.01$, $^{\#\#\#} p < 0.001$ compared to NC siRNA group. $^{\triangle} p < 0.05$, $^{\triangle\triangle} p < 0.01$, $^{\triangle\triangle\triangle} p < 0.001$ compared to plent-U6-GFP-Puro shRNA group, and ns = not statistically significant.

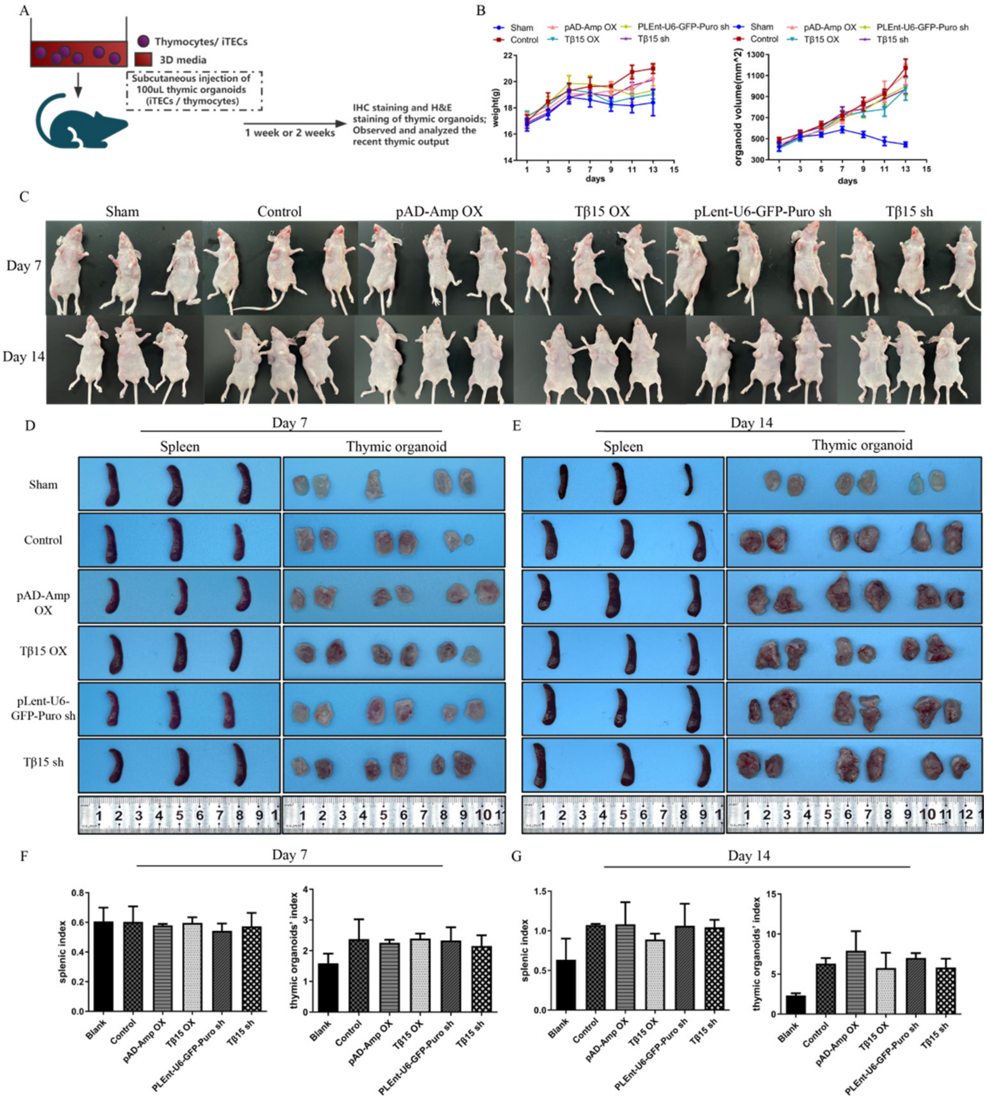

Supplementary Figure S2. An *in vivo* model of thymic organoids with thymocytes-iTECs interactions. (**A**) Workflow for subcutaneous transplantation of thymic organoids, showing that 100 µL thymic organoids are injected into bilateral foreleg axpits of 4-week-old female BALB/c nude mice (2×10^6 thymocytes and 1×10^5 iTECs per recipient mouse). 1 or 2 weeks after the transfer, recent thymic output function in the spleen and peripheral blood is analyzed with a flow cytometer, and

organoids are subjected to analysis of thymic development. (**B**) The trend of weight change (left) and the thymic organoids growth curve in nude mice after organoids transplantation. (**C**) Representative images of nude mice at 1 and 2 weeks after subcutaneous transplanted organoids, with n = 3 samples per condition. (**D-G**) Representative images and organ index of spleen and thymic organoids from the iTECs-specific Tβ15 altered (n = 3) and littermate control mice (n = 3) at week 1 (**D&F**) and 2 (**E&G**) of transplantation. Statistical analysis used was one-way ordinary ANOVA test followed by Tukey post-test. Data are mean ± SEM.

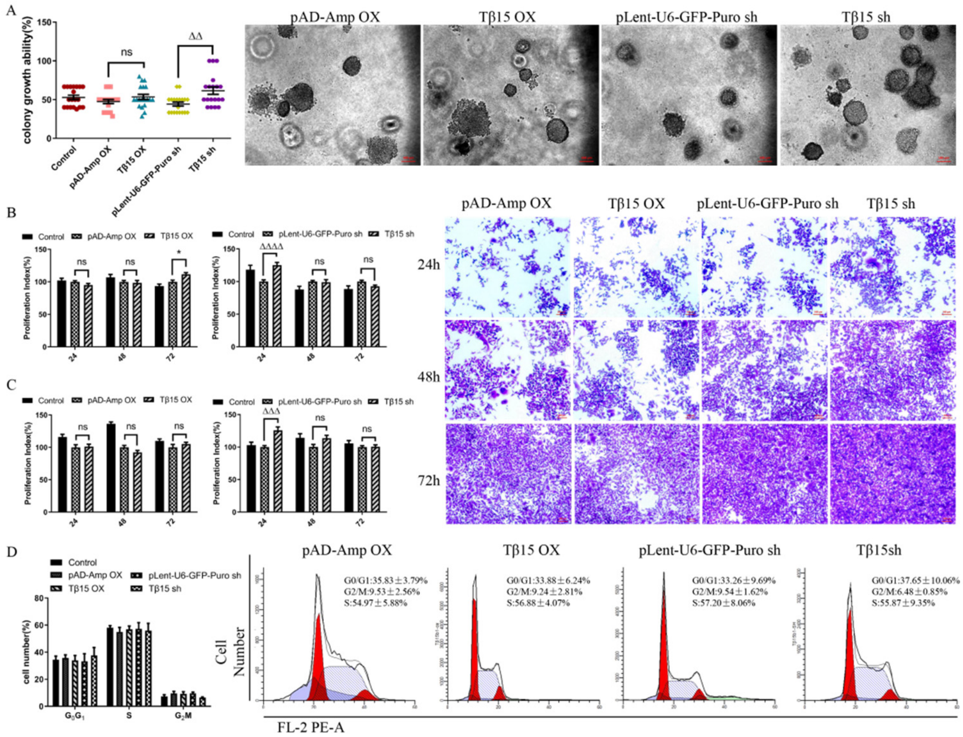

Supplementary Figure S3. Tβ15 overexpression causes the poor proliferative ability of iTECs. (**A**) Soft agar assays show that effect of Tβ15 gene overexpression/silencing on the colony growth ability of iTECs. Data represent the proportion of colonies with a diameter ≥100 μm formed in the soft agar assay. Scale bar = 100 μm. (**B&C**) MTT (upper panel) and crystal violet staining (lower panel) assess of the viability of iTECs treated with overexpression / silencing Tβ15 for different time. Scale bar = 100 μm. (**D**) Flow cytometry shows that effect of Tβ15 gene on different phases of iTECs cell cycle. The data represent the average percent cell populations in various phases of the cell cycle. All data are representative of three independent experiments, and graph shows mean ± SEM. A one-way ordinary ANOVA test was used in A, two-way ANOVA test in B-D. * $p < 0.05$, ** $p < 0.01$,*** $p < 0.001$ compared to pAD-Amp OX group. $^{\#} p < 0.05$, $^{\#\#} p < 0.01$, $^{\#\#\#} p < 0.001$ compared to NC siRNA group. $^{\triangle} p < 0.05$, $^{\triangle\triangle} p < 0.01$, $^{\triangle\triangle\triangle} p < 0.001$ compared to plent-U6-GFP-Puro shRNA group, and ns = not statistically significant.

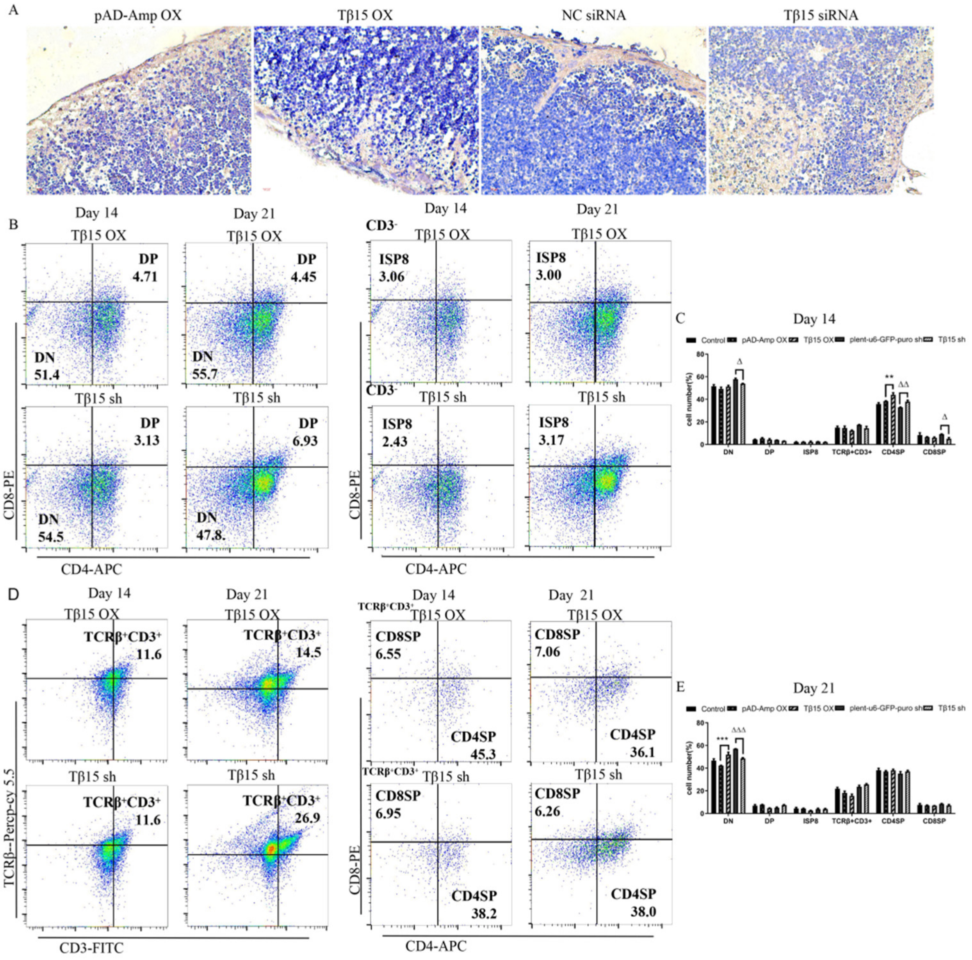

Supplementary Figure S4. Tβ15 affects the earliest stages of DN to DP development in organoids. (**A**) Representative images of TCRβ-positive staining at the rim of the thymus. Scale bar = 10 μm. (**B-E**) Comparison of T cell differentiation in thymic organoids formed in co-culture after 14, 21 days. Frequencies of DN cells ($CD3^{-}TCR\beta^{-}CD4^{-}CD8^{-}$), immature single-positive $CD8^{+}$ (ISP8) cells ($CD3^{-}TCR\beta^{-}CD4^{-}CD8^{+}$), double-positive (DP) cells ($CD4^{+}CD8^{+}$) and $TCR\beta^{+}$ $CD3^{+}$were shown as percentage of total cells. CD8SP cells ($CD3^{+}TCR\beta^{+}CD4^{-}CD8^{+}$) and CD4SP cells ($CD3^{+}TCR\beta^{+}CD4^{+}CD8^{-}$) were shown as percentage of $TCR\beta^{+}CD3^{+}$ cells in the thymic organoids. All data are representative of three independent experiments, and Graph shows mean ± SEM. Two-way ANOVA test was uesd in **C** and **E.*** $p < 0.05$, ** $p < 0.01$,*** $p < 0.001$ compared to pAD-Amp OX group. $^{\#}p < 0.05$, $^{\#\#}p < 0.01$, $^{\#\#\#}p < 0.001$ compared to NC siRNA group. $^{\triangle}p < 0.05$, $^{\triangle\triangle}p < 0.01$, $^{\triangle\triangle\triangle}p < 0.001$ compared to plent-U6-GFP-Puro shRNA group, and ns = not statistically significant..

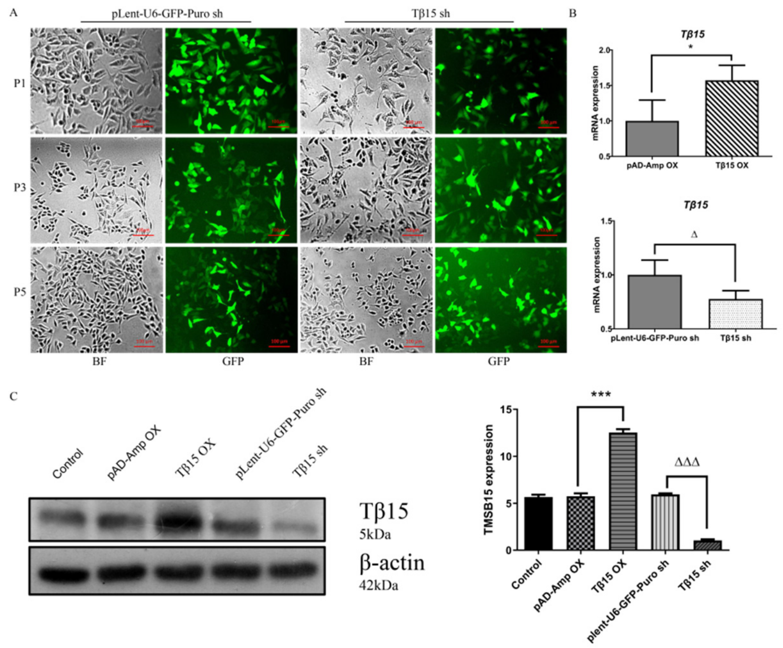

Supplementary Figure S5. The Tβ15 gene overexpression and knocking down efficacy in iTECs lines. (**A**) Representative images of iTECs-pLent-U6-GFP-Puro shRNA (**a**), iTECs-Tβ15 shRNA (**b**) at different passage numbers. Images are taken with respective channels for bright field (BF) and GFP. Scale bar = 100 μm. (**B**) Q-PCR shows the Tβ15 gene overexpression and knocking down efficacy in iTECs. Graph shows mean ± SEM. (**C**) Efficiencies of Tβ15 knockdown or overexpression in thymus were determined by Western blot. The expression level of TMSB15 is expressed as the ratio of the protein optical density value of each group to that of GAPDH. Nonpaired Student's t-test: $^{*}P < 0.05$ compared to pAD-Amp OX group. $^{\triangle}P < 0.05$ compared to pLent-U6-GFP-Puro shRNA group.

1.2. Supplementary Tables

Supplementary Table S1 Sequences of Tβ15 for quantitative real-time PCR

Gene name	Forward primers (5' - 3')	Reverse primers (5' - 3')
GAPDH	GGCTGCCCAGAACATCAT	CGGACACATTGGGGGTAG
Tβ15	CGGCAGACAA-GATGAGCGATA	ACCTTTGCAGCCAGGGTAGTA

Abbreviations: GAPDH, glyceraldehyde 3-phosphate dehydrogenase; Tβ15, thymosin beta 15.

Supplementary Table S2 Sequences of primer pairs for quantitative real-time PCR

Gene name	Forward primers (5' - 3')	Reverse primers (5' - 3')
TNF-α	TACAGCGACACTTGACACCC	TGCGGACCATAGAGAGTGGA

IL-1β	TGCCACCTTTTGACAGTGATG	GTGCTGCTGCGAGATTTGAA
IL-7	TCTGCTGCCTGTCACATCATC	GGACATTGAATTCTTCACTGA-TATTCA
Sema-3A	CAGCGCGTCTAGTGAGTGTT	CCCTCTCCCTGCTTCATTCG
Leptin	TTTCACACACGCAGTCGGTA	CACATTTTGGGAAGGCAGGC
ACTH	TGCTGTAATGAAGCCTCGCAG	GGCTCTTTGCAACCTCACAGA
FGFR2IIIb	CACGACCAAGAAGCCAGACT	CTCGGCCGAAACTGTTACCT
Foxn1	GACCTTGGGACTGACCTGGAT	TGCCTGTTTCTGCCAGACAA
Pax9	AATGCCATCCGCCTACGAAT	GATTTTGCTCACGCAGCCAT
DLL4	CTTCTTGCATAGGCGAGAACA	GGGTAAGATTTGGCGAACAGA
Ccl25	TTACCAGCACAG-GATCAAATGG	CGGAAGTAGAATCTCACAGCAC
β2M	TTCTGGTGCTTGTCTCACTGA	CAGTATGTTCGGCTTCCCATTC
L1cam	AGTTCCGCTGGACGAAAGATG	CGATAGATGCCCTGAAACCTCT
H2-K1	ACCAGCAGTACGCCTACGA	AACCAGAACAGCAACGGTCG
gimap3	TCATGCCGAGACACTCCTTAG	CAACACCTCCAACTTTTTGCC
gimap4	CGGGGTTCATCCCAGAAAGTT	CCCCAAGGATACTGTTCCCTG
Aire	AGGTCAGCTTCAGAGAAAAC	TCATTCCCAGCACTCAGTAGA
Fezf2	GTCACCGGCCACTTCTAAAAC	GTCTGCCTCTAACGCAGCA

Abbreviations: ACTH, adrenocorticotropin hormone; Aire, autoimmune regulator; β2M, beta2-microglobulin; Ccl25, chemokine ligand chemokine 25; Dll4, delta-like 4; Fezf2, forebrain embryonic zinc fingerlike protein 2; FGFR2IIIb, fibroblast growth factor receptor-2IIIb; Foxn1, forkhead-box n1; gimap3, GTPase immune-associated protein 3; gimap4, GTPase immune-associated protein 4; H2-K1, histocompatibility 2-K1; IL-1β, interleukin-1beta; IL-7, interleukin-7; L1cam, L1 cell adhesion molecules; Pax9, paired box gene 9; Sema-3A, semaphorin-3A; TNF-α, tumor necrosis factor-alpha.

1.3. Target Sequences of shRNA

Gene name	Target sequences
Ptmα-shRNA_1	GATCCGAGATCACCACCAAGGACTTGAATTCAAGAGATTCAAGTCCTT-GGTGGTGATCTCTTTTTTA
Ptmα-shRNA_2	GATCCGAGGCTGACAATGAGGTAGATGATTCAAGAGATCATCTACCTCATT-GTCAGCCTCTTTTTTA
Ptmα-shRNA_3	GATCCGATGGAGATGAAGATGAGGAAGCTTCAAGA-GAGCTTCCTCATCTTCATCTCCATCTTTTTTA
Ptmα-shRNA_4	GATCCGATGTGGACACCAAGAAGCAGAATTCAAGAGATTCTGCTTCTT-GGTGTCCACATCTTTTTTA
Tmsb15b1-shRNA_1	GATCCGTCGAATGAAACTATCCAGCAAGTTCAAGAGACTTGCTGGA-TAGTTTCATTCGACTTTTTTA
Tmsb15b1-shRNA_2	GATCCGACTTGTCAGAAGTTGAAACGTTCAAGAGAC-GTTTCAACTTCTGACAAGTCTTTTTTA
Tmsb15b1-shRNA_3	GATCCGCAACATCAATATTGCCTGACTTCAAGAGAGTCAGGCAA-TATTGATGTTGCTTTTTTA
Tmsb15b1-shRNA_4	GATCCGCTCCTGGTAGATCCATTTACTTCAAGAGAGTAAATGGATCTAC-CAGGAGCTTTTTTA

1.4. Target Sequences of adenovirus

1.4.1. >NM_008972.2 Mus musculus prothymosin alpha (Ptma), mRNA

CCGAAAAGCCATCTTTGCATTGTTCCTGGGTCGTGCTCCGCGCTCGCTGCAGCCAC-
CTTCGCCGCCCACCGCCTCCTCCAGCGCGGACTCCGGCAGCTCTCTCGCCAGAGTCCTCGA
ACTCGACTTTTAATTCCCTCACTCGCGGCATCGGACCACCGGCGTGCCCCAC-
CATGTCAGAC-
GCGGCAGTGGATACCAGCTCCGAGATCACCACCAAGGACTTGAAGGAGAAGAAGGAAGT
TGTGGAGGAGGCAGAGAATGGAAGAGATGCACCTGCCAATGGGAACGCTCAAAATGAG-
GAAAATGGGGAGCAGGAGGCTGACAATGAGGTAGATGAAGAAGAGGAAGAAGGTGGGG
AGGAAGAGGAGGAGGAGGAAGAAGGTGACGGTGAGGAGGAGGATGGAGATGAA-
GATGAGGAA-
GCTGAGGCTCCTACGGGCAAGCGGGTAGCTGAGGATGATGAGGATGACGATGTGGACAC
CAAGAAGCAGAAGACCGAGGAGGATGACTAGACAGCAAAAGGAAAAATAACCTTAC-
GCAC-
CGTGACCTATTCACCCTCCACTTCCCGTCTCAGAATTTAAACGTGGTCACCTTCGAGTAGA
GAAGCAGGCCCCGCCCGCCCACAGCGGGCAATGCCACCCACAGA-
TATGACATGCGCTCTCCACCACCCCTCCAAAACTACAACATGAATTGGCAACATGGGAGG
AAAAAAGAACCAGAACTTCCCAGGCCCTACTTTTTTTCTTAAAAA-
TACTTTAAAAGGAAAATTTGTTTGTATTTTTTATTTACATTTTATATTTTTGTACATATTGTTA
GGGGTCAGCCATTTTTAATGATAACGGGTGACCAAAC-
CAGCCTTCAGAGCGTTTTCTGTCC-
TACTTCAGACTTTACTTGTGGTGTGACCATGTTCATTATAATCTCAAAGGAGAAAAAAAAA
ACCTTGTAAAAAAAAGCAAAAACAACAACAAAAAAACAATCTTATTCCGAGCATTCCAG-
TAACTTTTGTGTATGTACCTAGCTGTACTATAAGTAGTTGGTTTGTATGAGATGGTTAAAAA
GGCCAAAGATAAAAAGATTTTTTTTTCCTTTTCTTTCTGTCTATGAAGTT-
GCTGTTTATTTTTTTTTGGCCTGTTTGATGTATGTGTGAAACAATGTCCAACAATAAACCGG
AATTTTATTTTGCTGAGTTGTTCTAAC

1.4.2. >NM_001081983.1 Mus musculus thymosin beta 15b1 (Tmsb15b1), mRNA

GGGGCGGGACCCGTGGCAGAGTTAATCAGTTGGTCACTGCACTCCTGAGCACAG-
TCCTGATCCAGAGGTAACTGTGGACATAACTGCTGGCCTGAAGGGAGCCTTGGAACCGGC
AGACAAGATGAGCGATAAACCAGACTTGTCAGAAGTTGAAACGTTT-
GACAAATCAAAGTT-
GAAGAAAACTAATACTGAAGTAAAAAATACTCTTCCGTCGAATGAAACTATCCAGCAAG
AGAAAGAACATAATGAAAGAACATAAAATGATACTCTCCTTTCAAGAGCAACATCAA-
TATTGCCTGACAGTCATGCCTCTAGGCTTGTTTCTATAACCCTATGCAATATATAGACATGT
TAGGCAGCTCCTGGTAGATCCATTTACTACCCTGGCTGCAAAGGTCAGCATTTGC-
TATGAATCATTAAATTTTTTTGGTACTGCTCA
